# Dealing with COVID-19 Epidemic in Italy: Responses from Regional Organizational Models during the First Phase of the Epidemic

**DOI:** 10.3390/ijerph18095008

**Published:** 2021-05-09

**Authors:** Maria Lucia Specchia, Andrea Di Pilla, Martina Sapienza, Maria Teresa Riccardi, Americo Cicchetti, Gianfranco Damiani, Instant Report Group

**Affiliations:** 1 Department of Life Sciences and Public Health, Università Cattolica del Sacro Cuore, Largo F. Vito 1, 00168 Rome, Italy; marialucia.specchia@unicatt.it (M.L.S.); martina.sapienza93@gmail.com (M.S.); mt.riccardi@gmail.com (M.T.R.); gianfranco.damiani@unicatt.it (G.D.); 2 Fondazione Policlinico Universitario “A. Gemelli” IRCCS, Largo A. Gemelli 8, 00168 Rome, Italy; 3 Postgraduate School of Health Economics and Management (ALTEMS), Università Cattolica del Sacro Cuore, Largo F. Vito 1, 00168 Rome, Italy; americo.cicchetti@unicatt.it

**Keywords:** healthcare system, community health services, healthcare organization, management, hospital, COVID-19, coronavirus, pandemic

## Abstract

As the COVID-19 outbreak traveled through various Italian regions, all national and local administrations issued measures to counter the spread of the contagion and organize healthcare. The Italian healthcare system is, indeed, a decentralized system with 21 regional health systems (RHSs), with different models of healthcare service delivery and organization. This study investigates whether a different organization of RHSs would have led to different management of the COVID-19 epidemic, and evaluates the effect of different approaches in epidemic management on the COVID-19 epidemiological trend. A set of indicators is identified by conducting an online synchronous Focus Group, involving an experts panel. A Pearson’s correlation test was performed on the values assumed by the historical series of indicators investigate correlations among the trends represented by the indicators or between them and external factors. The comparison between the experiences of the different Italian regions, regarding the management of the epidemic, has helped to confirm and emphasize the importance of a community-based approach in health care—integrated with the hospital’s functions for the care of complex conditions and the need for specialized assistance.

## 1. Introduction

Among European countries, Italy was the first to be affected by COVID-19. The first patients were detected in Lombardy and Veneto regions, on 21 February 2020. COVID-19, significantly different from seasonal influenza, spread rapidly, showing its dangerousness that was initially underestimated [1]. Notwithstanding, on 28 April 2020, the burden of COVID-19 in Italy, both in terms of mortality and morbidity, was estimated to be 121.449 disability-adjusted life years (DALYs), highlighting its magnitude. [2]. In this scenario, the healthcare workers role has been pivotal. In fact, they were exposed to an unknown threat, and they were both a high-risk category and a potential source of the outbreak [3,4].

In the last 40 years, previous outbreaks have taught us several things: Firstly, strong public health infrastructure and the existence of protocols at the national, intermediate, and community levels are necessary to avoid the spread of epidemics [5,6,7]. Secondly, community surveillance, the capacity for early detection, and the diffusion of correct information to the whole population are vastly important [8,9]. Thirdly, prepared healthcare services have become essential to respond in a proper way, to limit the spread of pathogens and the number of infected people [10].

Preparedness could be very different between national and regional levels [11,12,13]. When SARS-CoV-2 landed in Italy, there was little scientific literature on it—suggesting a viral spread that was expected to grow, challenging the preparedness and responsiveness of healthcare systems worldwide [14,15].

Italy has a National Health System (NHS) since 1978. During the last 20 years, many reforms led to a progressive decentralization of NHS [16]. Decentralization might have a positive effect a better integration among social and health care services, but during years several studies reported the increase of inequalities within the Italian country [17,18,19,20].

Under the Italian Constitution, responsibility for healthcare is shared by the central government, the 19 regions, and the two autonomous provinces. Each regional health system (RHS) is responsible for health care facilities and coordinates the local health authorities (LHAs), which organize and plan all the healthcare services in a given area [21]. 

The clinical severity of the first COVID patients suggested the need to strengthen the infectious disease and COVID-dedicated intensive care units, in analogy with what had already happened in China, to cope with the consequences of the spread of the epidemic [22].

As the epidemic progressed across the various Italian regions, all the national and local administrations issued measures to counter the spread of the contagion and to organize healthcare for patients. These measures ranged from social distancing, lockdown, quarantine, epidemiological and microbiological surveillance to the strengthening of health services at the national level, recruitment of the health staff, a supplement of the equipment and consumables, and the reorganization of territorial healthcare [23].

This study investigates whether a differences in the organization of regional health services would have led to different management of the COVID-19 epidemics, and evaluates the effect of different approaches in epidemic management on the COVID-19 epidemiological trend. To this end, the study proposes the following methodology, based on the available data.

## 2. Materials and Methods

### 2.1. Online Focus Group

This work was undertaken by a working group of the Università Cattolica del Sacro Cuore of Rome, composed of public health and health economics specialists from the Department of Life Sciences and Public Health and the High School of Health Economics and Management. To address the study aim, a set of indicators was identified by conducting an online synchronous Focus Group [24], involving an experts panel.

Synchronous online group, using conference calling or other online methods, is the closest approximation of classic face-to-face focus group and includes real-time debates headed by one or more moderators [24], allowing to overcome problems related to pandemic in-person meetings restrictive rules [25].

### 2.2. Expert Panel Selection

To assemble a panel of experts and key stakeholders that is easy to manage, numerous enough to get diversity of perspectives and limited enough not to become disorderly or fragmented, a number of ten participants is considered appropriate [26]. 

Therefore, the final expert panel consisted of 10 participants, including health economists, epidemiologists, public health experts in health systems research and organization, and statisticians belonging to the Department of Life Sciences and Public Health and to the High School of Health Economics and Management of the Università Cattolica del Sacro Cuore of Rome.

### 2.3. Approach

Two online focus groups were held via the Zoom online meeting platform (Zoom Video Communications, Inc., San Jose, CA, USA), which lasted approximately 90 min. The online meetings were led by a facilitator, who guided the group’s discussion, with the support of a notetaker from the study team [27].

### 2.4. First Synchronous Online Focus Group

The instruction and material were sent by mail the day before the focus group (Supplementary file S1).

At the beginning of the meeting, a moderator invited each expert to introduce himself and give some consideration about the project. After having discussed which indicators had to be considered the most proper ones, each panelist was asked to list in the free text at least four individual indicators, using the absolute data contained on the civil protection dataset. 

At the end of the first focus group, the duplicates were eliminated by the author group, and 17 remaining indicators were distributed by mail to all the panelists.

### 2.5. Second Synchronous Online Focus Group

The day before the second focus group, panelists received the instruction and material (Supplementary file S2). They were asked to assess the quality of each indicator by scoring if it was “relevant”, “strong”, “realistic”, and “achievable” [28]. During the focus group, participants discuss their preferences and independently vote whether to keep or discard each indicator, prioritizing indicators by choosing those they considered as most important and appropriate to describe the regional pandemic management organizational model. Results were collected by the moderators at the end of the session. Indicators approved by six or more panelists were included and constitute the final list of core indicators.

### 2.6. Monitoring Indicators

Data used to carry out the analysis were retrieved from the Official Website of the Protezione Civile [29]. Demographic data from the National Institute of Statistics (ISTAT) Official Website [30], and data from the Ministry of Health on the capacity of intensive care beds in the Italian regions were also used [31,32].

It was decided to follow weekly the evolution of epidemic and epidemic management at the regional level, through the indicators identified. The observation period lasted from 9 March 2020 (beginning of the lockdown throughout Italy) to 4 May 2020, the end of “Phase I” in managing the epidemic in Italy.

A timely daily report on a weekly basis was published to take a picture of the pandemic and to be able to follow its evolution over time. The daily values were elaborated by seven days weighted moving average, centered with lags of +/− three days, in order to minimize the accidental daily variability. On the basis of the weekly values assumed by the indicators identified, the 21 regions and autonomous provinces were divided into three orderly sections.

### 2.7. Correlation Analysis

A correlation test is used to estimate the relationship between two variables. Correlation is useful for quantifying how much of a phenomenon is a representation in relation to another, even though it is not necessarily a direct cause. Normal distributions were calculated using the Shapiro–Wilk test. A Pearson’s correlation test was performed to investigate indicators’ correlations to external factors or to the characteristics of the regional health system that existed prior to the epidemic or between themselves. The correlation test allowed the interpretation of the indicators taking into account the specific potential of the individual regions in the organization of healthcare and public health. The choice of the elements for which to test the correlation was made subordinately to identifying the indicators using the Focus Group.

## 3. Results

### 3.1. Core Indicators

Core Indicators implemented based on the Focus group methodology and published data are as follows:

1. Point prevalence in the Italian Regions (‰)
  This indicator represents the extent of contagion at the time of detection; the numerator gives the number of cases currently positive for the Sars-CoV-2 infection, while the denominator gives the regional population. The series of the values of Indicator 1 is shown in Table 1. On the basis of the data of point prevalence, it was possible to divide the 21 regions and autonomous provinces into three basic tertiles, at the beginning of the observation; regions with high prevalence were Piemonte, Lombardia, Valle d’Aosta, Veneto, Emilia-Romagna, Marche, P.A. of Trento (Table 1).
  Data show an evident north-south gradient in the spread of the contagion, which remained almost unchanged during the period of observation (Liguria and Provincia Autonoma (P.A.) of Bolzano risen among the regions with high prevalence, Lazio risen among the regions with intermediate prevalence, while Veneto fell from the high prevalence tertile to the intermediate one). Considering the average values for the period under examination, regions with the highest prevalence were: Lombardia, Piemonte, Emilia-Romagna, P.A. of Trento and Bolzano, Liguria, Marche, and Veneto (with the exception of Val d’Aosta, a small Special Statute Region).
2. Cumulative number of performed nasopharyngeal swabs/Resident population × 1000.
  This indicator stands for the regional testing policy; its values are shown in (Table 2).
  The use of shades of grey is, therefore, useful in interpreting the data based on their meaning rather than their value: The regions offering a limited number of tampons are in dark grey, just as in the previous table (Table 1) the regions with a high prevalence; thus, dark grey marks a possible criticality. Vice versa, in Table 2, the regions in white are those that were able to offer a greater number of diagnostic tests, just as in Table 1, the white regions were those with low prevalence, thus highlighting situations of minor criticality for the considered aspect.
  As regards Indicator 2, it is possible to see that Veneto, Friuli Venezia Giulia (FVG), Emilia Romagna, and P.A. of Trento are constantly placed in the first tertile, as well as the southern regions are mainly placed in the third tertile. With regard to the final value achieved by the Veneto region, first among the regions with the ordinary statute in Italy (and absolutely second only to the P.A. of Bolzano), it should be noted that Veneto has distinguished itself for a peculiar policy in the case research, offering the diagnostic test also to asymptomatic subjects since the beginning of the epidemic [33]. It was considered necessary by local decision-makers, since asymptomatic people were proved to transmit the Sars-CoV-2 virus [34].
  Considering the trend of diagnostic tests carried out in the group of the regions with infection high prevalence, we find the regions of Liguria and Piemonte among those that carried out the lowest number of diagnostic tests in relation to the population together with the Marche and Lombardia region, which is not only the largest region in Italy, but also the region with the highest prevalence of confirmed cases.
3. Saturation of Intensive Care Unit (ICU) beds.
  The indicator is calculated from the ratio between the number of COVID patients admitted to intensive care and the official number of beds available in ICUs before the outbreak of the epidemic: In Italy, before the epidemic, there were 5179 intensive care beds, an average of 8.6 beds per 100,000 inhabitants, with considerable regional variability (range: 11.8–5.9; SD: 1.5) [31]. This indicator highlights the pressure of COVID patients under serious clinical conditions on the healthcare system, in particular on hospital care, considering its capacities when the country was hit by the epidemic and how well prepared it was to withstand the shock; values are shown in Table 3.
  It can be noted that the north-south gradient already highlighted for the cumulative number of performed nasopharyngeal swabs is also present for this indicator. The regions Piemonte, Lombardia, and Marche are placed constantly in the first tertile of ICU beds saturation values; the Lombardia region, in particular, has reached 150% saturation (which is a theoretical value referring to the existing equipment, which does not take into account the expansion of the hospital network that had to be carried out in the course of the emergency), even if starting from an ICU bedding that was and still is the highest in the country—861 ICU beds before the outbreak of the epidemic, 8.6 per 100,000 inhabitants, perfectly in accordance with the national average. Conversely, among the regions with a high prevalence of infection, the saturation of ICU beds Veneto, Emilia-Romagna, and Liguria assume the lowest values, being among the regions with the highest standards of intensive care beds in Italy.
4. Currently hospitalized cases/currently confirmed cases
  This indicator expresses the propensity to treat patients in a hospital setting; values assumed by this indicator are shown in Table 4.
  In the case of this indicator, the north-south gradient is less evident. This indicator makes it possible to highlight an important difference in the management of COVID patients by the regions. Thanks to the division into tertile, it is, in fact, possible to identify three approaches: Hospital-centered approach for the regions in the first tertile (e.g., Piemonte), community-based approach for the regions the third tertile (e.g., Veneto), integrated approach for the regions in the second tertile (e.g., Emilia-Romagna).

### 3.2. Correlation Analysis

In the hypothesis that the possibility to have a community-based approach by regions is linked by its preparedness in primary care and community health services, the correlation between Indicator 4 and the most recent published data concerning the monitoring of essential health care levels (Livelli Essenziali di Assistenza, LEA) in the Italian regions [35] has been tested. In Italy, in fact, the Ministry of Health has been monitoring the performance of regions in three areas for a year: Hospital care, preventive medicine, and primary care and community health services. The latter area includes health services spread throughout the community, from general practitioners to pharmaceutical assistance, from specialist and outpatient diagnostics to home care and chronic conditions care. For primary care and community health services, some of the core indicators used by the Ministry of Health actually concern avoidable hospitalizations and home care [36]. Special Statute Regions Friuli Venezia Giulia, Val d’Aosta, Sardegna, P.A. of Trento and P.A. of Bolzano were not subject to verification of compliance with the LEA Committee [37].

The calculation of Pearson correlation coefficient between the score obtained for primary care and community health services (indicative of the ability to manage patients at home, avoiding hospitalizations) and an estimator of Indicator 4 (obtained through an average of daily values) for the regions subject to verification of the LEA requirements in the first tertile of prevalence gives a value of −0.453 (Table 5): For the regions with the highest prevalence, therefore, this indicator shows an inverse correlation with the score obtained by the respective regions on primary care and community health services. That is, the more regions were able to manage patients at home, avoiding hospitalizations (before the pandemic-demonstrating capability to offer and manage home care), the fewer COVID patients were admitted to hospitals (during the outbreak) and were able to be cared for at home.

Assuming that a hospital-centered or community-based approach by regions concerns both hospitalizations, and the number of diagnostic tests provided to the population, the correlation between Indicator 2 and the score obtained for primary care and community health services has been tested for the regions in the first tertile of prevalence subject to verification of compliance with the LEA Committee, giving a value of 0.521. This means that the greater the capacity of the regions in primary care and community health services, the greater the number of swabs that could be offered to the population. (Table 5).

**Figure 1:**
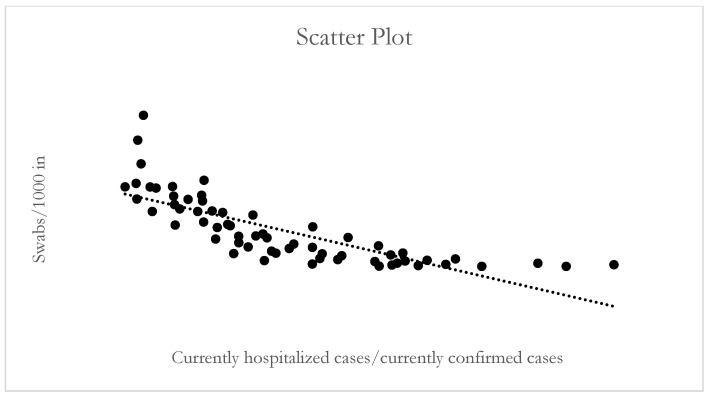
Indicator 4 and Indicator 2: Scatter plot.

In addition, to assess the consistency of the assumption that a community model is based both on less hospital management and a greater supply of swabs, the correlation between Indicator 4 and Indicator 2 has been tested in the regions with the highest prevalence subjected to verification of compliance with the LEA Committee. The inverse correlation (Pearson: −0.784; R^2^: 0.614) suggests that the less you can test the population, the more you need to refer cases to hospital facilities (rather than community) (Figure 1).

## 4. Discussion

The COVID-19 pandemic dominated scientific literature in 2020. [38] Many researchers try to describe the current situation by focusing on specific topics: For example, the situation of healthcare workers [3,39] or the predictive modeling to help health policies [40,41]. In Italy, several scientific papers were published, focusing on the clinical, epidemiological, and public health management of the disease in a specific region [42,43]. To our knowledge, there is no literature that used epidemiological data to give organizational photography of the different management of COVID-19 across Italian regions. The experience described in this paper has led to the definition of a set of indicators based on available open-source data, to evaluate the healthcare organizational aspects related to managing the COVID-19 epidemic in Italy and to identify the differences between the regional adopted approaches to make a constructive comparison [44].

It is firstly necessary to underline a significant geographical difference in the spread of the epidemic. Indicator 1 (point prevalence in the Italian regions) is, therefore, of fundamental importance: None of our results can be interpreted without taking into account the prevalence of cases in the different regional contexts. 

Indicator 2 (cumulative number of performed nasopharyngeal swabs/Resident population × 1000 inhabitants) is the first and clearest example of how prevalence is critical for interpreting other data. The number of tests carried out, in fact, in addition to the different approaches of the LHAs, strictly depends on the number of confirmed cases.

Another aspect concerns the availability of data, which represents both a strength and a limit of the indicators developed and monitored. This becomes particularly evident concerning Indicator 3 (saturation of Intensive Care Unit-ICU-beds on the days considered), whose numerator is taken from the data published daily by the surveillance system, but whose denominator has been defined from data of the Ministry of Health that have not followed the same daily evolution. In the high saturation regions, it was necessary to restructure hospital networks [22], and increase the number of ICU beds: The values of Indicator 3 do not, therefore, reflect a real situation, but a theoretical saturation that would have been obtained with the availability of beds present before the epidemic. The analysis of the dynamics of the expansion of the ICUs beds during the outbreak has not been the subject of in-depth study, for two main reasons. First, the preparedness of the system was our target. Second, there was regional heterogeneity in the availability of data. Conversely, Indicators 4 (currently hospitalized cases/currently confirmed cases) is the only indicator entirely fed by data published daily by the surveillance system, both as regards the numerator and the denominator, expressing an approach in managing patients.

Indicator 4 makes it possible to identify three different approaches corresponding to the tertiles of the indicator’s values, but, again, although this division is applicable to all 21 regions and P.A., it is essential to distinguish them based on prevalence. For the large regions of Northern Italy with the highest prevalence, it can be seen how Piemonte and Lombardia had hospital-centered management of the epidemic in the early stages, while Veneto had community-based management from the beginning and Emilia-Romagna opted for integrated management. It is interesting to note that regional differences in the standard of ICU beds available before the epidemic (a difference which may certainly have influenced differences in ICU bed saturation) do not appear to have translated into equivalent differences in patient management: The regions with the most ICU beds express different approaches (hospital-centered in Liguria, community-based in Veneto, integrated into Emilia-Romagna).

As shown by Pearson’s correlation coefficient between Indicator 4 and Indicator 2, a community-based or hospital-centered approach is not only seen in the proportion of positive hospitalized cases; it can also be identified in the provision of diagnostic tests to the population. In the community-based approach, not only the preferred setting of care was within the community, but the testing policy was wide.

The community-based approach has been promoted especially in the Veneto region: The model adopted at Vo Euganeo, in Veneto, consisting of lockdown and mass diagnostic tests, has proved capable of stopping the epidemic and has been reported and discussed in the scientific literature [43], and in the international press [45] confirming, however, that a significant proportion of confirmed cases of Sars-CoV-2 infections are asymptomatic and that, therefore, to stop the epidemic, it is not enough to intercept symptomatic patients in the hospital, leading to the need to develop innovative strategies to test cases, as it is impossible to rely solely on the onset of symptoms [46], since asymptomatic individuals nonetheless can infect others, and therefore, represent a critical element in public health actions to limit the epidemic [34]

The experiences of past epidemics have left us aware of the importance of sharing information at an early stage, especially for an epidemic caused by an unknown pathogen [47]. This is true not only for clinical information, but also with reference to the organizational models identified to face the epidemic, which may be more or less effective. This can be verified even if only by examining the data on the lethality of COVID-19, stratified by age classes [48]: Local differences that have emerged regarding the lethality of the virus are probably due to differences in case searching and differences in case management outside the hospital, both distinctive elements of the community-based approach [49].

It is, therefore, on the community-based approach that it is possible to identify, interpret and follow over time the differences in managing the epidemic and in its outcomes, and that approach is currently considered the most appropriate for dealing with epidemics and the COVID epidemic in particular, while a hospital-centered approach is considered not sufficient [50,51,52,53].

In fact, almost all the regions are witnessing a decrease in the value of Indicator 4, moving on to manage the majority of patients according to a community model (e.g., it can be seen how Lombardia progressively drops to 19.10% of cases managed in hospital). Within April 2020, all regions, except Lazio, are below the value that Indicator 4 assumed for the Veneto region at the beginning of the observation (33.67%), making probably inappropriate to interpret the final values of the indicator for Lombardia, Piemonte, Liguria, and Veneto (respectively 19.10%, 16.49%, 19.28%, and 14.49%), although belonging to different tertile, as expressions of different approaches.

With regard to the conditions that may have influenced the initial choice of regions to adopt one approach rather than another, it is interesting to note that Veneto—the only region in which the values of Indicator 4 are constantly in the third tertile (community-based approach)—is the region in Italy with the highest score for primary care and community health services. On the other hand, the three regions that are constantly in the first tertile (hospital centered approach) are Lombardy, Liguria and Lazio; the first two are regions with a high prevalence and are respectively the region of the North of Italy with the lowest score in terms of primary care and community health services (for those with verification of compliance) [35], and the region with the oldest population in Italy [54] (elderly COVID patients have a higher probability of hospitalization [55]. Lazio, on the other hand, has highly specialized hospital facilities that have been identified as COVID-Hospitals (INMI Spallanzani IRCCS and Fondazione Policlinico A. Gemelli IRCCS in primis, in addition to several other teaching hospitals and hospitals) and is also the region of the Center of Italy with the lowest score for primary care and community health services [35] (fourth-last, if we consider all the Italian regions subject to verification of compliance). 

Certainly, there is not a single factor that explains such differences. Decentralization brought the 21 Italian RHS to different starting points when the pandemic began [17,18,19,20].

Even in regions that are geographically adjacent and entirely comparable in socio-economic terms, there are decades of differences in regional and corporate health policies [56,57], which, for example, concern the more or less extensive presence of private providers in the regional health organization or the different relationships between commissioners and providers, but it is not the purpose of this study to go into in depth. [58,59].

It is worth focusing on the inverse correlation that emerged between the hospital-centered approach and the scores obtained for primary care and community health services, in regions with a high prevalence. Turning the point of view upside down, it is possible to state that the regions that had developed advanced facilities and experience on primary care and community health services and home care (traditionally associated with the care and management of chronic diseases) could quickly use them to cope with the epidemic. This suggests that primary care and community health services can be valuable for chronic, as well as community phenomena, such as an epidemic (insofar as the infection has a subacute course), in managing public health issues. In a context where the aging of the population and chronic conditions make it imperative to strengthen home care services and a progressive redefinition of the role of the hospital, the comparison between the experiences of the different Italian regions regarding managing the epidemic has helped to confirm and emphasize the importance of a community-based approach in health care, integrated with the hospital’s functions for the care of complex conditions and the need to receive highly specialized assistance [60].

Types of data are one of the limits of the work proposed in this paper: The data published daily by the surveillance system provide few types of information, insufficient for in-depth evaluations, however useful, on the evolution of contagions and on the quality and safety of health care. Additionally, data published on diagnostic tests contain the number of all the diagnostic tests daily processed, without distinguishing between diagnostic tests conducted to make an initial diagnosis and diagnostic tests conducted to follow the clinical course.

On the other hand, the strengths of this work include the scenarios opened up for research on the different healthcare approaches during the COVID-19 outbreak; in particular, the relationship between the approach (community or hospital-based) and the lethality of the infection should be the subject of dedicated studies.

The ability to adapt the monitoring of a phenomenon to the data published during an emergency, even if initially few in number, can be important for guiding both decisions, and the collection and publication of data in a logic of continuous improvement.

This work can represent a widely reproducible methodology, based on both qualitative and quantitative research methods, for the monitoring and evaluation of clinical and organizational management during epidemic emergencies with daily updates data. Compared to the daily reporting that is done by many researchers, this work represents an innovative attempt to investigate the reasons for regional differences in emergency management. The robustness of the results obtained, both in scientific terms and in the discussion of insiders on what models to follow and what measures to take in epidemic management (including the progressive improvement of data quality, according to a self-adaptive logic that has profitably fed the relationship between the surveillance system and the research on the data published by it), while being able to use few typologies of data, but having the opportunity to follow them over time, makes this experience transferable to other contexts and other countries.

## Figures and Tables

**Table 1:** Indicator 1: Point prevalence in the Italian regions (‰).

	09-Mar	16-Mar	23-Mar	30-Mar	06-Apr	13-Apr	20-Apr	27-Apr	04-Mag
Piemonte	0.08	0.34	1.06	1.78	2.42	2.95	3.39	3.60	3.57
Valle d’Aosta	0.11	0.81	2.85	4.16	4.64	4.58	4.25	1.83	0.88
Lombardia	0.42	1.09	1.88	2.50	2.81	3.16	3.42	3.53	3.59
Veneto	0.15	0.47	1.02	1.55	1.98	2.20	2.08	1.82	1.47
FVG	0.07	0.29	0.65	0.95	1.14	1.05	1.07	1.02	0.86
Liguria	0.07	0.38	1.00	1.56	2.04	2.22	2.28	2.31	2.28
Emilia-Romagna	0.28	0.70	1.58	2.40	2.89	3.06	3.00	2.69	1.99
Toscana	0.06	0.24	0.63	1.08	1.44	1.69	1.75	1.61	1.42
Umbria	0.04	0.20	0.65	0.99	1.00	0.74	0.48	0.33	0.21
Marche	0.22	0.78	1.55	2.14	2.39	2.07	2.12	2.19	2.12
Lazio	0.02	0.08	0.24	0.43	0.57	0.68	0.76	0.79	0.76
Abruzzo	0.02	0.14	0.47	0.89	1.11	1.37	1.58	1.56	1.42
Molise	0.05	0.06	0.17	0.37	0.60	0.67	0.70	0.66	0.60
Campania	0.02	0.06	0.16	0.30	0.47	0.54	0.53	0.50	0.46
Puglia	0.01	0.06	0.21	0.39	0.53	0.63	0.71	0.74	0.74
Basilicata	0.01	0.03	0.16	0.37	0.47	0.49	0.44	0.38	0.33
Calabria	0.01	0.05	0.15	0.31	0.38	0.42	0.44	0.41	0.36
Sicilia	0.01	0.04	0.14	0.29	0.37	0.42	0.46	0.44	0.45
Sardegna	0.01	0.06	0.22	0.38	0.51	0.56	0.53	0.48	0.41
P.A. Bolzano	0.05	0.47	1.27	1.99	2.36	2.81	2.90	1.77	1.20
P.A. Trento	0.07	0.64	1.66	2.48	3.37	3.81	3.54	3.00	2.09

Table 1: values in the first tertile (high prevalence regions) are in dark grey; Values in the second tertile (regions with intermediate prevalence) are in light grey; Values in the third tertile (low prevalence regions) are blank; FVG stands for Friuli Venezia Giulia.

**Table 2:** Indicator 2: Cumulative number of performed nasopharyngeal swabs/Resident population × 1000.

	09-Mar	16-Mar	23-Mar	30-Mar	06-Apr	13-Apr	20-Apr	27-Apr	04-Mag
Piemonte	0.43	1.30	3.21	5.97	9.73	16.03	23.43	32.52	41.10
Valle d’Aosta	0.53	2.63	8.48	12.35	20.74	29.18	38.31	52.23	66.34
Lombardia	2.03	4.31	7.33	11.11	15.45	20.95	27.08	34.38	41.86
Veneto	3.45	7.05	12.73	20.64	30.22	41.68	53.66	65.96	78.86
FVG	1.12	3.54	6.33	11.78	18.79	25.90	38.41	51.24	63.31
Liguria	0.40	1.49	3.61	6.40	10.31	15.23	21.56	28.94	36.85
Emilia-Romagna	1.13	2.94	6.97	12.01	16.25	22.20	29.19	37.32	45.14
Toscana	0.56	1.63	3.95	8.32	14.59	21.90	28.90	35.35	41.75
Umbria	0.25	1.33	4.20	9.32	14.73	21.65	29.67	38.17	45.39
Marche	0.82	2.13	4.51	7.30	10.54	17.26	28.39	35.23	43.49
Lazio	0.45	1.58	3.13	5.54	8.38	12.46	16.83	21.78	26.74
Abruzzo	0.20	1.23	2.99	6.34	11.03	16.09	22.04	26.81	32.16
Molise	0.60	0.95	1.79	3.24	5.39	8.36	12.67	18.74	24.49
Campania	0.18	0.43	1.01	2.30	4.29	6.43	8.92	12.05	15.78
Puglia	0.18	0.62	1.73	3.35	5.52	8.09	11.20	14.49	17.15
Basilicata	0.23	0.41	1.26	3.31	5.51	8.22	12.64	19.02	26.87
Calabria	0.12	0.60	2.16	4.70	7.19	9.82	12.98	17.01	20.96
Sicilia	0.18	0.56	1.34	3.03	4.82	7.59	10.77	14.67	18.12
Sardegna	0.11	0.51	1.63	3.05	4.68	6.94	9.56	13.46	17.58
P.A. Bolzano	0.14	3.55	11.36	19.97	31.61	44.84	58.37	72.08	84.16
P.A. Trento	0.59	2.48	6.15	11.94	20.81	33.48	46.71	60.53	77.13

Table 2: values in the first tertile (high number of swabs) are blank; Values in the second tertile (regions with intermediate number of swabs) are in light grey; Values in the third tertile (low number of swabs) are in dark grey.

**Table 3:** Indicator 3: Saturation of intensive care unit (ICU) beds.

	09-Mar	16-Mar	23-Mar	30-Mar	06-Apr	13-Apr	20-Apr	27-Apr	04-Mag	ICU Beds/10^5^ in
Piemonte	16.93%	57.74%	103.75%	136.87%	133.60%	114.68%	91.44%	65.75%	49.75%	7.6
Valle d’Aosta	0.00%	38.75%	197.50%	259.38%	208.13%	146.25%	87.50%	60.63%	25.00%	8.0
Lombardia	51.59%	95.80%	135.82%	154.19%	152.59%	132.08%	103.16%	78.98%	60.57%	8.6
Veneto	11.08%	31.22%	56.84%	71.20%	63.17%	48.48%	36.01%	24.72%	20.24%	10.1
FVG	2.45%	15.00%	37.29%	49.74%	38.85%	23.49%	18.80%	10.99%	3.65%	9.9
Liguria	10.73%	42.53%	76.25%	95.69%	89.65%	75.31%	54.97%	44.55%	37.33%	11.8
Emilia-Romagna	19.21%	43.85%	62.22%	76.36%	81.68%	73.91%	63.70%	53.19%	42.79%	10.1
Toscana	6.07%	32.20%	61.55%	75.82%	73.01%	61.23%	49.06%	40.39%	28.94%	10.1
Umbria	3.93%	22.95%	55.18%	64.82%	62.32%	53.93%	38.04%	24.02%	16.96%	8.0
Marche	42.01%	92.99%	126.90%	145.27%	124.62%	96.14%	70.92%	49.40%	37.61%	7.6
Lazio	1.98%	6.09%	15.38%	26.94%	34.14%	34.98%	32.46%	26.01%	16.48%	9.9
Abruzzo	2.90%	25.36%	42.89%	57.27%	54.12%	41.92%	30.64%	18.34%	11.33%	9.5
Molise	7.92%	15.42%	22.92%	27.92%	17.71%	13.33%	8.96%	3.33%	2.71%	10.0
Campania	2.05%	6.77%	34.61%	38.49%	31.12%	24.63%	18.32%	11.70%	7.85%	5.9
Puglia	1.36%	3.89%	15.69%	33.84%	37.81%	22.88%	20.48%	16.12%	12.69%	7.7
Basilicata	0.38%	4.34%	22.58%	35.59%	36.35%	24.49%	15.18%	13.14%	6.38%	8.9
Calabria	0.51%	5.22%	13.31%	12.71%	9.80%	8.60%	4.67%	4.49%	2.53%	7.7
Sicilia	0.19%	4.96%	14.22%	17.39%	17.30%	12.65%	9.33%	8.07%	6.49%	8.6
Sardegna	0.00%	1.63%	13.15%	18.19%	19.40%	19.03%	15.95%	13.76%	7.56%	8.3
P.A. Bolzano	2.53%	27.36%	92.74%	155.91%	157.94%	115.54%	58.78%	39.86%	28.72%	6.9
P.A. Trento	7.42%	59.18%	151.76%	237.70%	245.12%	176.17%	121.48%	73.63%	50.39%	5.9

Table 3: values in the first tertile (high saturation regions) are in dark grey; Values in the second tertile (regions with intermediate saturation) are in light grey; Values in the third tertile (low saturation regions) are blank; The right column represents the standard of intensive care beds per 100,000 inhabitants that the region had before the epidemic.

**Table 4:** Indicator 4: Currently hospitalized cases/currently confirmed.

	09-Mar	16-Mar	23-Mar	30-Mar	06-Apr	13-Apr	20-Apr	27-Apr	04-Mag
Piemonte	81.52%	89.10%	56.00%	45.93%	37.61%	29.59%	24.03%	19.41%	16.49%
Valle d’Aosta	16.89%	28.82%	24.26%	22.23%	21.35%	22.14%	20.57%	38.60%	61.45%
Lombardia	77.03%	63.97%	55.62%	51.77%	46.94%	41.33%	31.84%	23.86%	19.10%
Veneto	33.67%	28.80%	29.62%	26.20%	20.24%	15.55%	14.12%	13.59%	14.49%
FVG	22.29%	33.81%	28.70%	24.59%	16.66%	15.17%	12.52%	11.64%	12.50%
Liguria	68.15%	62.44%	59.48%	53.70%	41.29%	34.09%	28.26%	23.10%	19.28%
Emilia-Romagna	53.89%	51.18%	42.84%	38.33%	32.31%	27.86%	25.39%	23.71%	24.10%
Toscana	51.88%	41.27%	45.30%	34.81%	25.93%	19.52%	15.90%	13.45%	11.58%
Umbria	22.91%	29.07%	25.48%	24.82%	23.29%	25.76%	32.14%	36.36%	37.95%
Marche	58.06%	54.75%	42.47%	35.50%	31.10%	33.44%	27.06%	21.57%	13.33%
Lazio	66.78%	63.97%	56.93%	49.63%	43.51%	38.52%	35.81%	35.03%	32.59%
Abruzzo	77.85%	63.28%	46.10%	34.46%	28.22%	22.07%	17.45%	16.93%	16.77%
Molise	41.56%	54.85%	59.93%	32.80%	20.96%	15.52%	13.47%	10.07%	5.61%
Campania	37.62%	35.53%	42.73%	35.38%	26.19%	22.00%	21.03%	19.99%	17.92%
Puglia	57.78%	54.57%	40.01%	44.47%	34.86%	27.13%	22.90%	17.28%	14.77%
Basilicata	33.72%	30.53%	29.16%	25.18%	24.62%	27.08%	27.86%	29.42%	28.86%
Calabria	70.69%	48.80%	36.39%	25.25%	26.03%	21.74%	17.64%	15.57%	14.34%
Sicilia	32.26%	46.01%	43.01%	39.06%	34.81%	29.54%	24.97%	21.77%	18.37%
Sardegna	37.71%	32.23%	25.53%	21.54%	17.99%	14.94%	15.47%	14.52%	15.15%
P.A. Bolzano	39.60%	26.36%	27.95%	28.31%	26.25%	15.78%	11.93%	16.21%	17.85%
P.A. Trento	42.41%	31.84%	34.39%	31.54%	23.48%	18.27%	15.74%	13.14%	12.80%

Table 4: in the table, the values of the regions in the first tertile are highlighted in dark grey and indicate the regions with the highest number of hospitalized patients compared to the total number of confirmed cases; the values in the third tertile are highlighted in white and those in the second in light grey.

**Table 5:** The score for primary care and community health services and the Indicators 2 and 4: Pearson correlation coefficient.

	Primary Care and Community Health Services Score	Swabs/1000 in	Currently Hospitalized Cases/Currently Confirmed Cases
Piemonte	88.31	14.86	44.41%
Lombardia	83.44	18.28	45.72%
Veneto	94.65	34.92	21.81%
Liguria	86.84	13.87	43.31%
Emilia-Romagna	94.32	19.24	35.51%
Marche	76.7	16.63	35.25%
Toscana	89.79	17.44	28.85%
*Pearson*		0.521	−0.453

## Data Availability

The study was conducted using opendata on the Regions provided by the Civil Protection, available at https://github.com/pcm-dpc/COVID-19/tree/master/dati-regioni (accessed on 18 December 2020).

## Supplementary Materials

The following are available online at https://www.mdpi.com/article/10.3390/ijerph18095008/s1, Supplementary file S1: Instant Report on COVID-19 situation in Italian Regions. Focus Group: 1st session; Supplementary file S2: Instant Report on COVID-19 situation in Italian Regions. Focus Group: 2nd session.
