# Supplementary material for: Dealing with COVID-19 Epidemic in Italy: Responses from Regional Organizational Models during the First Phase of the Epidemic
Source: Int J Environ Res Public Health. 2021 May 9;18(9):5008. doi: 10.3390/ijerph18095008 (PMC8125969; doi:10.3390/ijerph18095008)
Supplement: Supplementary file 1 [file ijerph-18-05008-s001.zip › ijerph-1174353-supplementary.pdf]

Supplementary material: worksheets FOCUS GROUP #1:

INSTANT REPORT

ON COVID-19 SITUATION IN ITALIAN REGIONS

FOCUS GROUP

1st session

Dear Colleague,

We are in the middle of a storm.

Among European Countries, Italy was the first to be affected by COVID-19, and vigilance, preparedness, speed and transparency have been identified as crucial to afford it. Regional differences exist, becoming everyday more evident in dealing with this new threat: are our Regions prepared? Will our Regional Health Systems be speed and transparent?

The "Protezione Civile" is publishing daily data (at National and Regional level) about the pandemic. Our aim is to achieve a better understanding of the preparedness of our Regional Health Systems. Even if we will face the limitation associated with reporting in real time the evolution of an emerging disease, we would like to try to do it in the best of our possibilities, using indicators that reflect the point and daily values represented by the available data.

You are asked to complete the following sheets with four indicators that, in your opinion, could be useful to the aim of our Instant Report.

Please, specify the numerator and the denominator per each of them.

INDICATOR #1

notes

INDICATOR #2

notes

INDICATOR #3

notes

INDICATOR #4

notes

Thanks for completing your task.

Supplementary material: worksheets FOCUS GROUP #2:

INSTANT REPORT

ON COVID-19 SITUATION IN ITALIAN REGIONS

FOCUS GROUP

2nd session

Dear Colleague,

Thanks for your contribution to the first online focus group. The defined indicators are collected here.

You are asked to assess each of them independently by evaluating if they are: relevant, strong, realistic, and achievable.

At the end of your evaluation, please give us an overall judgement by ticking the “yes” or “no” box. As discussed in the first session, if possible, choose “yes” when an indicator have ALL of the four characteristics mentioned before.

INDICATOR #1

$$\frac{\text{\# of currently hospitalized patient in ICU}}{\text{\# of currently hospitalized patients}} \times 100$$

Given the aim of the Report, do you think this indicator is:

Relevant ☐

Strong ☐

Realistic ☐

Achievable ☐

Would you include this indicator in the final analysis?

YES ☐

NO ☐

INDICATOR #2

$$\frac{\text{\# of currently hospitalized patient in ICU}}{\text{\# of ICU beds available*}} \times 100$$

*before the Sars-CoV2 outbreak

Given the aim of the Report, do you think this indicator is:

Relevant ☐

Strong ☐

Realistic ☐

Achievable ☐

Would you include this indicator in the final analysis?

YES ☐

NO ☐

INDICATOR #3

$$\frac{\text{\# of currently hospitalized patients}}{\text{\# of currently home-confined patients}} \times 100$$

Given the aim of the Report, do you think this indicator is:

Relevant ☐

Strong ☐

Realistic ☐

Achievable ☐

Would you include this indicator in the final analysis?

YES ☐

NO ☐

INDICATOR #4

$$\frac{\text{\# of new daily positive patients}}{\text{Regional population}} \times 100$$

Given the aim of the Report, do you think this indicator is:

Relevant ☐

Strong ☐

Realistic ☐

Achievable ☐

Would you include this indicator in the final analysis?

YES ☐

NO ☐

INDICATOR #5

$$\frac{\text{Tot. \# of deaths}}{\text{Regional population}} \times 100$$

Given the aim of the Report, do you think this indicator is:

Relevant ☐

Strong ☐

Realistic ☐

Achievable ☐

Would you include this indicator in the final analysis?

YES ☐

NO ☐

INDICATOR #6

$$\frac{\text{Tot. \# of deaths}}{\text{Tot. \# of confirmed cases}} \times 100$$

Given the aim of the Report, do you think this indicator is:

Relevant ☐

Strong ☐

Realistic ☐

Achievable ☐

Would you include this indicator in the final analysis?

YES ☐

NO ☐

INDICATOR #7

$$\frac{\text{\# of currently hospitalized patient in ICU}}{\text{\# of currently confirmed cases}} \times 100$$

Given the aim of the Report, do you think this indicator is:

Relevant ☐

Strong ☐

Realistic ☐

Achievable ☐

Would you include this indicator in the final analysis?

YES ☐

NO ☐

INDICATOR #8

$$\frac{\text{\# of currently positive patients}}{\text{Regional population}} \times 100$$

Given the aim of the Report, do you think this indicator is:

Relevant ☐

Strong ☐

Realistic ☐

Achievable ☐

Would you include this indicator in the final analysis?

YES ☐

NO ☐

INDICATOR #9

$$\frac{\text{\# of currently hospitalized patients}}{\text{Tot. \# of currently positive patients}} \times 100$$

Given the aim of the Report, do you think this indicator is:

Relevant ☐

Strong ☐

Realistic ☐

Achievable ☐

Would you include this indicator in the final analysis?

YES ☐

NO ☐

INDICATOR #10

$$\frac{\text{\# of total performed NP swabs}}{\text{Resident population}} \times 1.000$$

Given the aim of the Report, do you think this indicator is:

Relevant ☐

Strong ☐

Realistic ☐

Achievable ☐

Would you include this indicator in the final analysis?

YES ☐

NO ☐

INDICATOR #11

$$\frac{\text{Tot. \# of total confirmed patients}}{\text{Tot. \# of total performed NP swabs}} \times 100$$

Given the aim of the Report, do you think this indicator is:

Relevant ☐

Strong ☐

Realistic ☐

Achievable ☐

Would you include this indicator in the final analysis?

YES ☐

NO ☐

INDICATOR #12

$$\frac{\text{\# of currently home-confined patients}}{\text{\# of currently positive patients}} \times 100$$

Given the aim of the Report, do you think this indicator is:

Relevant ☐

Strong ☐

Realistic ☐

Achievable ☐

Would you include this indicator in the final analysis?

YES ☐

NO ☐

INDICATOR #13

$$\frac{\text{\# of recovered patients}}{\text{Tot. \# of confirmed patients}} \times 100$$

Given the aim of the Report, do you think this indicator is:

Relevant ☐

Strong ☐

Realistic ☐

Achievable ☐

Would you include this indicator in the final analysis?

YES ☐

NO ☐

INDICATOR #14

$$\frac{(\text{\# of currently hospitalized patient}) - (\text{\# of currently hospitalized patient in ICU})}{\text{\# of currently positive patients}} \times 100$$

Given the aim of the Report, do you think this indicator is:

Relevant ☐

Strong ☐

Realistic ☐

Achievable ☐

Would you include this indicator in the final analysis?

YES ☐

NO ☐

INDICATOR #15

$$\frac{\text{Tot. \# of deaths}}{\text{Tot. \# hospitalized patient}} \quad \text{X 100}$$

Given the aim of the Report, do you think this indicator is:

Relevant ☐

Strong ☐

Realistic ☐

Achievable ☐

Would you include this indicator in the final analysis?

YES ☐

NO ☐

INDICATOR #16

$$\frac{\textbf{Tot. \# of deaths}}{\textbf{Tot. \# home-confined patients}} \quad \textbf{X 100}$$

Given the aim of the Report, do you think this indicator is:

Relevant ☐

Strong ☐

Realistic ☐

Achievable ☐

Would you include this indicator in the final analysis?

YES ☐

NO ☐

INDICATOR #17

$$\frac{\text{\# of currently hospitalized patients}}{\text{\# of currently confirmed cases}} \times 100$$

Given the aim of the Report, do you think this indicator is:

Relevant ☐

Strong ☐

Realistic ☐

Achievable ☐

Would you include this indicator in the final analysis?

YES ☐

NO ☐

Thanks for completing your task.

Supplementary material: OUTPUT OF FOCUS GROUP #2:

	# 1	# 2	# 3	# 4	# 5	# 6	# 7	# 8	# 9	# 10	# 11	# 12	# 13	# 14	# 15	# 16	# 17
Expert 1	x	v	v	x	v	x	v	v	v	x	x	x	x	x	x	x	v
Expert 2	v	v	v	x	x	x	v	v	x	v	v	x	x	x	x	v	v
Expert 3	x	x	v	x	v	x	v	v	v	v	x	x	x	v	x	x	x
Expert 4	v	v	x	x	x	x	v	v	v	v	x	x	v	x	x	v	v
Expert 5	x	x	x	x	x	x	x	x	x	x	x	x	x	x	x	x	v
Expert 6	v	v	v	x	v	x	v	v	x	v	v	v	x	x	x	x	v
Expert 7	x	x	x	v	x	x	x	x	x	v	x	x	x	v	x	x	v
Expert 8	x	v	x	v	x	v	x	v	x	v	x	x	x	x	x	v	v
Expert 9	v	x	x	v	x	v	x	x	x	v	x	x	x	v	x	x	v
Expert 10	x	v	x	x	x	v	x	v	x	v	x	x	x	x	x	x	v
